# Long Noncoding RNA Plays a Key Role in Metastasis and Prognosis of Hepatocellular Carcinoma

**DOI:** 10.1155/2014/780521

**Published:** 2014-03-16

**Authors:** Guangbing Li, Haohai Zhang, Xueshuai Wan, Xiaobo Yang, Chengpei Zhu, Anqiang Wang, Lian He, Ruoyu Miao, Shuguang Chen, Haitao Zhao

**Affiliations:** ^1^Department of Liver Surgery, Peking Union Medical College Hospital, Chinese Academy of Medical Sciences and Peking Union Medical College (CAMS & PUMC), Beijing 100730, China; ^2^Department of Liver Transplantation and Hepatobiliary Surgery, Provincial Hospital Affiliated to Shandong University, Jinan, Shandong 250021, China; ^3^Department of General Surgery, Peking Union Medical College Hospital, Chinese Academy of Medical Sciences & Peking Union Medical College, Beijing 100730, China

## Abstract

Long noncoding RNAs (lncRNAs) have been attracting immense research interests. However, only a handful of lncRNAs had been thoroughly characterized. They were involved in fundamental cellular processes including regulation of gene expression at epigenetics as well as tumorogenesis. In this paper, we give a systematic and comprehensive review of existing literature about lncRNA involvement in hepatocellular carcinoma. This review exhibited that lncRNAs played important roles in tumorigenesis and subsequent prognosis and metastasis of hepatocellular carcinoma and elucidated the role of some specific lncRNAs such as MALAT1 and HOTAIR in the pathophysiology of hepatocellular carcinoma and their potential of being therapeutic targets.

## 1. Introduction

Human genome only composes about 25,000 protein-coding genes as published by the International Human Genome Sequencing Consortium [1]. The remaining bigger portion of human genome was not functional and being considered as “junk DNA” [2, 3]. Studies have explored these “junk DNA” based on RNA deep sequencing and genome-wide analysis that the “junk DNA” was not derived from any known genes and does not encode any protein [4, 5]. Most of the “junk DNA” is intron DNAs [6], which are also called noncoding DNA (ncDNA) [7]. Some ncDNAs are transcribed into functional noncoding RNA (ncRNA), while the others are either not transcribed or transcribed to RNA of unknown function.

ncRNAs are classified into small ncRNAs and long ncRNAs (lncRNAs) based on size. Small ncRNAs include siRNAs, piRNAs, and miRNAs that have a length of less than 200 nucleotides (nt). lncRNAs are greater than 200 nt in length, frequently up to 100 kb [5]. Many types of ncRNA do have known biological functions, such as transcriptional and translational regulation of protein-coding sequences [8, 9].

lncRNAs are located in nuclear or cytosolic fractions. They are usually transcribed by RNA polymerase II but have no open reading frame and map to intronic and intergenic regions. Moreover, lncRNAs display epigenetic features common to protein-coding genes, such as trimethylation of histone 3 lysine 4 (H3K4me3) at the transcriptional start site (TSS) and trimethylation of histone 3 lysine 36 (H3K36me3) throughout the gene body. It has been estimated that nearly 15,000 lncRNAs are presented in the human genome, but only a small fraction is expressed in a given cell type [10, 11].

lncRNAs were initially thought to be the product of a “noisy” inconsequential transcription resulting from low RNA polymerase fidelity [12]. Recent studies have demonstrated that lncRNAs regulated several biological processes such as transcription, translation, cellular differentiation, gene expression regulation, cell cycle regulation, chromatin modification, and nuclear-cytoplasmic trafficking [13–15]. Tripathi et al. found that lncRNA MALAT1 modulated expression of cell cycle genes and was required for G1/S and mitotic progression [16]. Li et al. found that the Hox transcript antisense intergenic RNA (HOTAIR) induced PTEN methylation, thus promoting human laryngeal squamous cancer cell proliferation [17].

## 2. Role of lncRNA in Cancer

### 2.1. Mechanism of Cancer Development

Cancer is a genetic disease—a result of dysregulation of genomic networks [18]. Despite extensive study, the majority of the genetic components of cancer susceptibility have not been linked to individual genes [19, 20]. Exploration of the role of regulatory elements variation, such as ncDNA, in gene expression may become a key development in exploring the molecular mechanisms of cancer.

The ncDNA serves not only as a substrate for DNA-binding proteins that in turn control both the expression and 3D architecture of the genome but also as a template for transcription of vast numbers of ncRNAs [21]. Both the small ncRNAs and lncRNAs play a central role in regulating cellular activities in Eukaryotes. The alteration and dysregulation of several ncRNAs have been reported in several types of human cancers [22].

### 2.2. Effect of lncRNA on Cancer

The mechanisms through which lncRNA contributes to the cancer development are diverse. Evidences suggested that one of the major roles of lncRNA was to guide the site specificity of chromatin-modifying complexes to affect epigenetic changes [21]. lncRNA could regulate gene expression at transcriptional and posttranscriptional level by targeting either local or distant genes [23]. Recently, lncRNAs have also shown their tumorigenic potential by modulating transcription of p53 [24]. A 3 kb lncRNA, linc-RNA-p21, transcriptionally activated by p53, collaborated with p53 in order to control gene expression in response to DNA damage. Silencing of linc-RNA-p21 depresses the expression of hundreds of genes through interaction with heterogeneous nuclear rib nucleoprotein K (hnRNP-K), thus promoting apoptosis of abnormal cells or restraining tumors [25].

Dysregulated expression of lncRNA in cancer marks the spectrum of disease progression and may serve as an independent predictor for patient outcomes. Dr. Han et al. conducted lncRNA and mRNA profile comparison between glioblastoma and normal brain tissue. Their results indicated that the lncRNA expression profile in glioblastoma tissue was significantly altered and associated with recurrence and malignant progression of GBM [26]. Dr. Shu et al. found that a new lncRNA GAS6 antisense RNA 1 (lncRNA GAS6-AS1) was downregulated in non-small-cell lung cancer, and its expression served as an independent predictor for overall survival. Low expression of lncRNA GAS6-AS1 was associated with poor prognosis [27]. Results of Jia's team showed that overexpression of long noncoding RNA PCAT-1 was a novel biomarker of poor prognosis in patients with colorectal cancer [28].

### 2.3. Long Noncoding RNA as Oncogene

Cancer is a genetic disease. Epigenetic events play key roles in promoting tumor initiation and progression. Chromosomal instability is a common molecular event in cancer development and is associated with cancer invasiveness and metastasis [29]. lncRNAs mediate epigenetic changes by recruiting chromatin remodeling complexes to specific genomic loci. lncRNA may serve as oncogene in cancer development and progression. Several examples illustrated the silencing potential of lncRNAs [30]. A recent study found that 20% of 3300 human lncRNAs were bound by polycomb-repressive complex 2 (PRC2) [31]. A lncRNA X-inactive-specific transcript (XIST), which is encoded by XIST gene, recruits the chromatin regulator PRC2 to this chromosome and promotes the formation of heterochromatin through histone modifications. Dysfunction of XIST may trigger the chromatin instability and promote caner development.

Another important effect of lncRNAs on chromatin modification in cancer was exemplified by the lncRNA, antisense noncoding RNA in the INK4 locus (ANRIL). It controls the epigenetic status of the INK4b/ARF/INK4a locus by interacting with subunits of PRC1 and PRC2. High expression of ANRIL has been found in certain cancer tissues such as melanoma and prostate cancers [32].

Many literatures show that hundreds of lncRNAs are sequentially expressed in the human homeobox (Hox) loci [33]. Transcription of these lncRNAs could demarcate chromosomal domains of gene silencing [34]. And overexpression of these lncRNAs can accelerate cancerogenesis. HOX antisense intergenic RNA (HOTAIR) regulates HOXD gene expression through induction of repressive chromatin state [11]. Overexpression of HOTAIR was found in breast and colon cancers and was associated with metastasis and poor prognosis [30, 35, 36].

Another lncRNA serving as oncogene is retinal noncoding RNA 2 (RNCR2). Rapicavoli et al. found that RNCR2 plays a critical role in regulating mammalian retinal cell proliferation. Knockdown of RNCR2 resulted in an increase of both amacrine cells and Muller glia [37]. Also, lncRNA CCAT1 may be an oncogene. Level of lncRNA CCAT1 was markedly increased in gastric carcinoma tissue comparing with normal tissue, and overexpressed CCAT1 promoted cancer cell proliferation and migration [38].

The lncRNA, metastasis-associated lung adenocarcinoma transcript 1 (MALAT1), is a highly conserved nuclear ncRNA. It acts as a molecular decoy serving as a structural link in rib nucleoprotein (RNPs) complexes. Ji and colleagues developed a MALAT1 knockout model in human lung tumor cells [39]. They found that MALAT1 did not alter alternative splicing but rather actively regulated gene expression including a set of metastasis-associated genes. Consequently, MALAT1-deficient cells were impaired in migration and formed fewer tumor nodules in mouse xenograft. Antisense oligonucleotides (ASO) that block MALAT1 prevented metastasis formation after tumor implantation.

Natural antisense transcripts (NATs) are a large class of lncRNA transcribed from the antisense DNA strand to other transcripts and overlap in part with sense RNA. NATs can exert their regulatory functions by acting as epigenetic regulators of gene expression and chromatin remodeling [40]. NATs have been implicated in several processes such as RNA translation and transcriptional interference, where they play a pivotal role in cancer. Alpha hypoxia-inducible factors (*α*HIF), which are derived from the 3′ UTR of HIF1, represent the first case of overexpression of a NAT associated with a specific human malignant disease: non-papillary clear-cell renal carcinomas. Moreover, it has been demonstrated that *α*HIF expression was a poor prognosis marker in breast cancer [41]. The ANRIL mentioned above also is an antisense lncRNA originating from the INK4B-ARF-INK4A locus, which contains three tumor suppressor genes. ANRIL was found to be overexpressed in prostate cancer tissues. Repression of ANRIL expression was associated with a reduction in cellular proliferation and increased the expression of both p16 Ink4A and p15 INK4B, which are encoded by CDKN2A and CDKN2B, respectively [42].

BOKAS is a natural antisense transcript of Bok, a proapoptotic member in the Bcl-2 family. The expression of BOKAS was found in testis and certain cancer tissues but not in other normal adult tissues. Overexpression of BOKAS was able to inhibit Bok-induced apoptosis in HeLa cells [43].

Another example of NAT is Zeb2/Sip1, which regulates E-cadherin expression by increasing the level of Zeb2 protein, a transcriptional repressor of E-cadherin. This finding suggested a role of ncRNAs in the control of epithelial morphology [44, 45].

### 2.4. Long Noncoding RNA as Tumor Suppressor

In addition to these lncRNAs acting as oncogenes, there are also lncRNAs with tumor suppressor function. One well-known example is the lncRNA Growth Arrest-Specific 5(GAS5) [46]. It was originally identified in mouse NIH3T3 fibroblasts [47, 48]. GAS5 binds to the DNA-binding domain of the glucocorticoid receptor (GR) and acts as a decoy glucocorticoid response element (GRE), thus competing with DNA GREs [49]. GAS5 negatively regulates the survival of lymphoid and breast cells and is aberrantly expressed in several cancers [50]. Pickard et al. showed that GAS5 promotes apoptosis of prostate cells after irradiation with UV, and low GAS5 expression therefore reduces the effectiveness of chemotherapeutic agents [24, 51].

Recent studies have unveiled other properties of lncRNAs. For instance, lncRNAs can regulate mRNA stability. One example is the tumor suppressor pseudogene PTENP1. The 3′ UTR region of this gene is very similar to the untranslated region of PTEN transcript [52]. Both of these regions bind to the same set of miRNAs. PTENP1 pseudogene belongs to the group of competing endogenous RNAs (ceRNAs). It may act as “decoy” by protecting PTEN mRNA from binding to common miRNA and therefore allowing expression of the tumor suppressor protein. Similarly, KRAS and KRAS1P transcript levels were found to be positively correlated, corroborating that pseudogene functions mirror the role of their cognate genes as explained by the miRNA decoy mechanism. Specific mutations at the binding site of these pseudogenes impair their activity, thus promoting tumor progression.

## 3. Role of lncRNA in Hepatocellular Carcinoma

Hepatocarcinogenesis is a complex process associated with accumulation of genetic and epigenetic changes that occur during initiation, promotion, and progression of the disease [53]. Abnormal lncRNAs expression can influence genes associated with hepatocarcinogenesis [22]. Recently, many studies focused on the contributions of lncRNAs to HCC development, revealing that differential expression of lncRNAs played critical roles in hepatocarcinogenesis, microvascular invasion, and metastasis [54–58].

Studies showed that lncRNAs play important roles in cell cycle control [59, 60], which is one of the molecular mechanisms in cancerogenesis [61]. Dr. Yang and his colleagues found that one lncRNA, named lncRNA-HEIH, was overexpressed in HCC tissue compared with normal liver tissues using microarray. Downregulation of lncRNA-HEIH induces *G*(0)/*G*(1) arrest that may be caused by the interaction of lncRNA-HEIH with enhancer of zeste homolog 2 (EZH2). lncRNA-HEIH increases the binding of EZH2 levels, thus influencing expression of EZH2 target genes. Their study showed that the expression level of lncRNA-HEIH in hepatitis B virus- (HBV-) related HCC was significantly associated with recurrence of tumor and was an independent prognostic factor for survival [62].

lncRNAs may also participate in the HBV-related hepatocarcinogenesis. Hepatitis B virus X protein (HBx) has been implicated as an oncogene in both epigenetic modifications and genetic regulation during hepatocarcinogenesis [63]. Huang et al. identified one lncRNA, named HBx-related long noncoding RNA (lncRNA-Dreh) that was downregulated by HBx protein [58]. lncRNA-Dreh could bind to the intermediate filament protein vimentin, repress its expression, and thus change the cytoskeleton structure and inhibit tumor metastasis [58]. It acts as a tumor suppressor in the development of HBV-HCC, which inhibits HCC growth and metastasis* in vitro* and* in vivo*. These findings support a role of lncRNA-Dreh in tumor suppression and survival prediction of HCC patients.

Angiogenesis in HCC is one of the risk factors for HCC metastasis [64]. A novel lncRNA was found to be associated with microvascular invasion in HCC, named lncRNA-associated microvascular invasion in HCC, lncRNA-MVIH [65]. Dr. Yuan et al. found that lncRNA-MVIH could promote tumor growth and intrahepatic metastasis by contributing to active angiogenesis both* in vitro* and* in vivo* through the inhibition of phosphoglycerate kinase 1 (PGK1) secretion [65].

A lncRNA, highly upregulated in liver cancer (HULC), was found to contribute to tumorigenesis of HCC [66, 67]. HULC was characterized as a novel mRNA-like ncRNA presenting in the cytoplasm as well as plasma [68] and playing an important role in posttranscriptional modulation of gene expression [69]. Depletion of HULC resulted in a significant deregulation of several genes involved in liver cancer. Fine tuning of HULC expression is part of an autoregulatory loop in which inhibition of expression and activity of microRNA, miR-372, allows lncRNA upregulated expression in liver cancer [67]. In HBV-related liver cancer, HBx induces upregulation of HULC, which in turn suppresses the expression of p18 and facilitates proliferation of HCC [70]. HULC is specifically increased in blood and tumor tissue of HCC patients and has the potential to be a biomarker. Higher HULC expression was found to be positively correlated with Edmondson histological grades or with HBV positive status [69, 71].

HOTAIR is a lncRNA that was identified from the HOXC locus (12q13.13) [30]. HOTAIR forms a complex with the polycomb-repressive complex 2 (PRC2), composed of EZH2, SUZ12, and EED, and binds to trimethylate histone H3 at lysine 27 (H3K27me3), thereby inhibiting HOXD gene expression [72]. Studies have demonstrated that HOTAIR can reprogram chromatin state to promote cancer metastasis [30]. Upregulation of HOTAIR is associated with metastasis of gastric cancer, lung cancer, and esophageal squamous cell carcinoma [73–77]. It is also a prognosis biomarker of esophageal squamous cell carcinoma [74] and overexpressed in hepatocellular carcinoma [78]. Ishibashi et al. found that HOTAIR was overexpressed in 13 out of 64 HCC patients [79]. Patients with HOTAIR expression had significantly bigger primary tumor sizes and poorer prognoses than those without HOTAIR expression.

Mineral dust-induced gene (MDIG), a lncRNA, was first identified in chronic lung diseases resulting from occupational exposure to mineral dust in the mining industry. MDIG was independently identified in human glioblastoma cell line T98G cells. The expression of MDIG is regulated by the c-Myc oncogene and named as myc-induced nuclear antigen 53 (mina53) [80]. Dysfunction of MDIG was found in several types of solid cancers including gastric carcinoma [81], esophageal squamous cell carcinoma [82], and lung cancer [83]. Overexpression of MDIG was observed in hepatocellular carcinoma [84]. Ogasawara et al. detected the expression of MDIG in 53 surgically resected HCC tissues through immunohistochemistry. Their results showed that MDIG was expressed in the nuclei of cancer cells in the tumor nodule and MDIG expression was high in the tumors larger than 2 cm in diameter than in those smaller than 2 cm. Also, MDIG expression was higher in poorly differentiated HCC than in well-differentiated HCC [85].

H19 is an imprinted, maternally expressed oncofetal gene. Studies have identified H19 as an oncogene [86, 87]. Overexpression of H19 was found in hepatocellular carcinoma. Ariel et al. detected that H19 was overexpressed in 13 of 18 HCC cases. Their results suggested that H19 might be used for histopathological and cytological diagnosis of hepatocellular carcinoma [88].

Metastasis-associated lung adenocarcinoma transcript 1 (MALAT1) is a lncRNA of 7 kb that is involved in cell growth and cell cycle progression [89]. Overexpressed MALAT1 was found in many solid tumors such as lung cancer, cervical cancer, and HCC [39, 90, 91]. Dr. Lai and his team have evaluated the expression of MALAT1 by quantitative real-time PCR in 9 liver cancer cell lines and 112 HCC patients. The results of their study showed that MALAT1 was upregulated in both cell lines and clinical tissue samples. MALAT1 was suggested to be an independent prognostic factor for predicting HCC recurrence. Patients with high expression level of MALAT1 had a significantly increased risk of tumor recurrence [92].

Maternally expressed gene 3 (MEG3) is a human homolog of mouse Gtl2. MEG3 is highly expressed in the normal human pituitary, including normal gonadotrophic cells [93]. Expression of MEG3 in tumor cells results in growth suppression, p53 protein increase, and activation of p53 downstream targets. MEG3 expression is lost in human gonadotroph-derived pituitary adenomas and most human tumor cell lines. Dr. Zhang and his colleagues found that the expression of MEG3 is associated with pathogenesis and progression of meningioma [94]. Dr. Huang and his team found that MEG3 is downregulated in HCC compared to normal liver tissues [95]. Expression of MEG3 may be regulated by microRNA-29 [96].

## 4. Conclusions

Cancer is widely perceived as a heterogeneous group of disorders with markedly different biological properties. Researches proved that cancers were caused by a series of clonally selected genetic changes in key tumor-suppressor genes and oncogenes. Dysfunction of lncRNA plays key role in cancerogenesis as shown in Table 1. Abnormal expression of lncRNA might interrupt gene expression in genetic and epigenetic level and was associated with prognosis of hepatocellular. This implied the possibility of lncRNA to become a therapeutic target of liver cancer.

## Figures and Tables

**Table 1:** Long noncoding RNA expressed in the HCC.

LncRNA ID	Dysregulation	Upstream regulators	Downstream targets	Cellular functions	Clinicopathological features
lncRNA-Dreh	Downregulated	HBx protein	Vimentin	Cytoskeleton structure	Prognosis
lncRNA HEIH	Upregulated		EZH2	Cell cycle	Prognosis
			PRC2		
lncRNAMVIH	Upregulated		PGK1	Microvessel growth	Metastasis
					Prognosis
HULC	Upregulated	CREB	miR-372	Proliferation	Metastasis
		HBx	p18		
HOTAIR	Upregulated	Suz-Twelve	PRC2	Chrome state	Metastasis
			LSD1		Prognosis
MDIG	Upregulated	c-Myc and RB	H3K9me3	DNA repair	Prognosis
			IGF2		
			macrosatellite X56		
			Jhdm3a		
MALAT1	Upregulated	TGF-beta	Caspase-3	Proliferation	Metastasis
			Caspase-8	Apoptosis	Prognosis
			BAX	Migration	
			BCL-2	Invasion	
			BCL-XL	Synaptogenesis	
			PRC1		
MEG3	Downregulated	cAMP	p53	Proliferation	Prognosis
			MDM2		
			GDF15		
